# Integer Representation of Decimal Numbers for Exact Computations

**DOI:** 10.6028/jres.111.006

**Published:** 2006-04-01

**Authors:** Javier Bernal, Christoph Witzgall

**Affiliations:** National Institute of Standards and Technology, Gaithersburg, MD 20899, USA

**Keywords:** computational geometry, Delaunay triangulation, exact integer arithmetic, power diagram, regular triangulation, robustness, Voronoi diagram

## Abstract

A scheme is presented and software is documented for representing as integers input decimal numbers that have been stored in a computer as double precision floating point numbers and for carrying out multiplications, additions and subtractions based on these numbers in an exact manner. The input decimal numbers must not have more than nine digits to the left of the decimal point. The decimal fractions of their floating point representations are all first rounded off at a prespecified location, a location no more than nine digits away from the decimal point. The number of digits to the left of the decimal point for each input number besides not being allowed to exceed nine must then be such that the total number of digits from the leftmost digit of the number to the location where round-off is to occur does not exceed fourteen.

## 1. Introduction

Mainstream computers base integer and floating point arithmetic on fixed word lengths. As a consequence, only values with a limited number of significant digits can be represented directly, so that the results of arithmetic operations may have to be rounded off or truncated. Such errors can be avoided or, at least, mitigated, by implementing special algorithms for the execution of arithmetic operations. A fully “exact arithmetic”, however, would have to be based on quotients of integers for representing numerical values. In any case, a final limitation is due to finite memory.

The need for exact arithmetic became apparent during the development of software for generating triangular and tetrahedral nets from very large point sets. Typically, this need is not due to high accuracy requirements for results—the input data are often noisy or given up to only a few significant digits—but is rather due to the need to maintain the consistency of a combinatorial structure. Building or manipulating such geometry-based combinatorial structures requires the calculation of indicators such as determinants in order to evaluate their sign and to check for zero values: round-off may lead to a false sign or zero value. An example considered later in this paper is to decide whether four given spatial points are coplanar. The approach of using exact computations for implementing computational geometry algorithms in a robust manner has been addressed in [2], [3], [4], [5], [6], [7]. Some computational geometry implementations ([2], [3], [4], [5]) reduce computational effort by utilizing exact arithmetic selectively whenever a decision might be affected by round-off.

In this paper, we document software for exact integer arithmetic, accommodating an indeterminate number of digits, for multiplication, addition, subtraction, but excluding division. We found that, in many computational geometry applications, decision variables such as determinants can be calculated without division. Also the sign of a decision variable stated as a quotient but not evaluated is readily derived from the signs of the numerator and denominator.

We also describe a preprocessing step, called “two-integer decomposition”, which leads from floating point input to one composed of integers only. At the root of this step lies the concept of space as an integer grid of points, all of which have integer coordinates in some shared unit. After completing the transition from floating point numbers to intermediate representations as pairs of integers—prompted by the fact that Fortran 77 does not provide a double precision integer format—a polynomial decomposition creates the number representations to be used in the exact arithmetic calculations. Software for this preprocessing step together with software for exact integer arithmetic has been successfully incorporated into several computational geometry related programs such as REGTET [1].

In what follows, a “standard computer” is a computer that uses 64 bits of storage for a double precision number and 32 bits for an integer. Given a standard computer, even though it may not store exactly an input decimal number as a double precision floating point number, it is safe to assume that the number will be represented as accurately as possible by a double precision floating point number up to its fourteenth significant digit.

## 2. Two-Integer Decomposition

Let *x*(*i*), *i* = 1, …, *n*, be a double precision array into which input numbers *x_i_*, *i* = 1, …, *n*, have been read. The two-integer decomposition process is a preprocessing step that takes place before any computations based on the input data are carried out. It rounds off the numbers in the array at a prespecified location of their decimal fractions and decomposes each rounded off number into two integers that are saved in integer arrays, say *ix*(*i*), *ix*2(*i*), *i* = 1, …, *n*. The rounded off numbers are then saved in array *x*(*i*), *i* = 1, …, *n*.

Given integers *k*, *l*, 1 ≤ *k* ≤ 9, 0 ≤ *l* ≤ 9, *k* + *l* ≤ 14, and assuming each input number *x_i_*, *i* = 1, …, *n*, has no more that *k* digits to the left of the decimal point, each number *x*(*i*), *i* = 1, …, *n*, is rounded off at the *l*th digit of its decimal fraction and decomposed into two integers in one of two ways according to its size. If the absolute value of *x*(*i*) times (10.0*d*0)*^l^* is less than 2^30^(=1073741824), *x*(*i*) is multiplied by (10.0*d*0)*^l^* and rounded off at the decimal point. The resulting integer is then placed in *ix*(*i*) while *ix*2(*i*) is set to zero. Finally, *x*(*i*) is redefined to be the double precision value of integer *ix*(*i*) divided by (10.0*d*0)*^l^*. On the other hand, if the absolute value of *x*(*i*) times (10.0*d*0)*^l^* exceeds or equals 2^30^, *x*(*i*) is truncated at the decimal point. The resulting integer (absolute value less than 2^30^ since *k* ≤ 9) is placed in *ix*(*i*). In addition, the signed decimal fraction obtained by subtracting the double precision value of this integer from the initial value of *x*(*i*) is multiplied by (10.0*d*0)*^l^* and rounded off at the decimal point. The resulting integer (absolute value less than 2^30^ since *l* ≤ 9) is placed in *ix*2(*i*). Next, *x*(*i*) is redefined to be the double precision value of integer *ix*(*i*) plus the value obtained by dividing the double precision value of integer *ix*2(*i*) by (10.0*d*0)*^l^*. Finally, if the integer *ix*2(*i*) is zero then *ix*2(*i*) is set to 2^30^ so that *ix*2(*i*) is zero if and only if the initial absolute value of *x*(*i*) (before the two-integer decomposition process) times (10.0*d*0)*^l^* is less than 2^30^.

The following is Fortran code for carrying out the two-integer decomposition process. Variables are either integer or double precision following convention. It is noted that while some loss in precision may occur at the time the input numbers are read and transformed into double precision floating point numbers, some additional loss in precision may occur here as well when the decimal point in a number is shifted by dividing or multiplying it by a multiple of 10.0*d*0, when the signed decimal fraction of a number is obtained by truncating the number at its decimal point and subtracting the result from the initial value of the number, and when a number is rounded off with the two-integer decomposition process. However once the two-integer decomposition process is completed all computations that follow, exact and otherwise, are carried out in terms of the arrays *x*(*i*), *ix*(*i*), *ix*2(*i*), *i* = 1, …, *n*, under the assumption that for the purposes of the user for each *i*, *i* = 1, …, *n*, *x*(*i*) represents closely enough the input number *x_i_* rounded off at the *l*th digit of its decimal fraction, and an integer (not necessarily stored by the computer) in terms of *ix*(*i*) and *ix*2(*i*) represents closely enough the input number *x_i_* times 10*^l^* rounded off at the decimal point.


  mfull=1073741824
  if(l.lt.0 .or. l.gt.9) stop 10
  isclu = 1
  dscle = 1.0d0
  if(l.eq.0) go to 200
  do 100 i = 1, l
  isclu = 10*isclu
  dscle = 10.0d0*dscle
100 continue
200 continue
  dfull = dble(mfull)
  dfill=dfull/dscle
  do 300 i = 1, n
  ix2(i) = 0
  if(dabs(x(i)).lt.dfill) then
    ix(i) = idnint(dscle*x(i))
   if(iabs(ix(i)).lt.mfull) then
     x(i) = dble(ix(i))/dscle
    go to 300
   endif
  endif
  if(dabs(x(i)).ge.dfull) stop 20
  ix(i) = idint(x(i))
  if(iabs(ix(i)).ge.mfull) stop 30
  decml = (x(i) - dint(x(i)))*dscle
  ix2(i) = idnint(decml)
  if(iabs(ix2(i)).eq.0) then
   x(i) = dble(ix(i))
   ix2(i) = mfull
  else
   x(i) = dble(ix(i)) + (dble(ix2(i))/dscle)
  endif
300 continue


## 3. Polynomial Decomposition

Given an integer *l*, 0 ≤ *l* ≤ 9, let *x_i_*, *i* = 1, …, *n*, be input numbers whose double precision floating point representations have been rounded off at the *l*th digit of their decimal fractions through the two-integer decomposition process. Let *x*(*i*), *ix*(*i*), *ix*2(*i*), *i* = 1, …, *n* be the arrays produced by the two-integer decomposition process that contain the rounded off numbers and the two-integer decompositions. For each *i*, *i* = 1, …, *n*, an integer *J*(*i*, *l*) is symbolically defined as follows (its actual value is not necessarily computed or stored by the computer). If *ix*2(*i*) equals zero then *J*(*i*, *l*) is set equal to *ix*(*i*). If *ix*2(*i*) equals 2^30^ then *J*(*i*, *l*) is set equal to *ix*(*i*) · 10*^l^*. Finally, if *ix*2(*i*) is neither zero nor 2^30^ then *J*(*i*, *l*) is set to *ix*(*i*) · 10*^l^* + *ix*2(*i*). In all cases for each *i*, *i* = 1, …, *n*, *J*(*i*, *l*) is considered to approximate closely enough (for the purposes of the user) the input number *x_i_* times 10*^l^* rounded off at the decimal point.

Set *M* to 2^15^. Given *J*(*i*, *l*), 1 ≤ *i* ≤ *n*, the polynomial decomposition process is a procedure (presented below in the form of Fortran subroutine decmp2) that decomposes the integer *J*(*i*, *l*) into a unique collection of integers *isga*, *isga* in {−1, 0, 1}, *ika*, *ika* > 0, *a_k_*, 0 ≤ *a_k_* < *M*, *k* = 1, …, *ika*, such that *J*(*i*, *l*) equals *isga* $\left(\sum_{k=1}^{\text{ika}}a_{k}\cdot M^{k-1}\right)$, *isga* the sign of *J*(*i*, *l*). Integers *a_k_*, *k* = 1, …, *ika*, are saved in an integer array, say *ia*(*k*), *k* = 1, …, *ika*, and the collection of integers *isga*, *ika*, *ia*(*k*), *k* = 1, …, *ika*, and the symbolic expression *isga* $\left(\sum_{k=1}^{\text{ika}}ia\left(k\right)\cdot M^{k-1}\right)$ are then called, respectively, the polynomial decomposition and the symbolic polynomial representation of *J*(*i*, *l*), with *isga* as the sign of the representation. For each *i*, *i* = 1, …, *n*, the polynomial decomposition of the integer *J*(*i*, *l*) is identified each time an exact computation involving additions, subtractions, or multiplications is required that references the input number *x_i_*. During one such computation, for each *i*, 1 ≤ *i* ≤ *n*, if the number *x_i_* is referenced in the computation, once the polynomial decomposition of the corresponding integer *J*(*i*, *l*) is identified, each reference of *x_i_* in the computation is replaced by the symbolic polynomial representation of *J*(*i*, *l*). The computation then takes effect as a sequence of additions, subtractions, or multiplications of symbolic polynomial representations with the final result being itself the symbolic polynomial representation of some integer. This final result can usually be used in only one of two ways. If it is known that for some positive integer *m* the integer that is equal to the final symbolic polynomial representation is approximately equal to the product of (10*^l^*)*^m^* and the true value of the computation, then this integer is computed approximately as a double precision floating point number from its symbolic polynomial representation and the true value of the computation is then approximately obtained by dividing it by ((10.0*d*0)*^l^*)*^m^*. On the other hand, if the purpose of the computation is simply that of obtaining the sign of the true result then the sign of the final symbolic polynomial representation is a satisfactory answer.

The concepts of polynomial decomposition and symbolic polynomial representation defined above for *J*(*i*, *l*), 1 ≤ *i* ≤ *n*, can also be defined for any integer *K* (not necessarily stored by the computer) in the same manner. Accordingly, the following is a Fortran subroutine called decomp for finding the polynomial decomposition *isga*, *ia*(*k*), *k* = 1, 2, (*ika* is already known to equal 2) of an integer *iwi* (stored by the computer) with absolute value less than 2^30^. Here *mhalf* equals 2^15^(=32768).


subroutine decomp(ia, isga, iwi, mhalf)
integer ia(*), isga, iwi, mhalf, ivi
if(iwi.gt.0) then
 isga = 1
 ivi = iwi
elseif(iwi.lt.0) then
 isga =-1
 ivi = -iwi
else
 isga = 0
 ia(1) = 0
 ia(2) = 0
 return
endif
ia(2) = ivi/mhalf
ia(1) = ivi - ia(2)*mhalf
return
end


In particular if *isclu* is set to 10*^l^* then *isclu* is less than 2^30^ (since *l* ≤ 9) so that the polynomial decomposition *isgu* (equal to 1), *iu*(*i*), *i* = 1, 2, (*iku* is already known to equal 2) of *isclu* can be obtained by calling subroutine decomp with a Fortran instruction as follows.


call decomp(iu, isgu, isclu, mhalf)


Finally, the following is a Fortran subroutine called decmp2 for finding the polynomial decomposition *isga*, *ika*, *ia*(*k*), *k* = 1, …, *ika*, of the integer *J*(*i*, *l*), 1 ≤ *i* ≤ *n*. Here *iwi* equals *ix*(*i*), *iwi*2 equals *ix*2(*i*), *mhalf* equals 2^15^, *mfull* equals 2^30^, and *iu*(*k*), *k* = 1, 2, is an array such that the polynomial decomposition of 10*^l^* is *iu*(1), *iu*(2) (isgu and iku are already known to equal 1 and 2, respectively). In addition, it is assumed that subroutines mulmul and muldif (presented below) exist for multiplying and subtracting, respectively, two symbolic polynomial representations.


  subroutine decmp2(ia, isga, ika, iwi, iwi2, mhalf, mfull, iu)
  integer nkmax
  parameter (nkmax=5)
  integer ia(*), isga, ika, iwi, iwi2, mhalf, mfull, iu(*)
  integer ie(nkmax), io(nkmax), isge, isgo, ike, iko, isgu, iku
  call decomp(ia, isga, iwi, mhalf)
  ika = 2
  if(iwi2.ne.0) then
   isgu = 1
   iku = 2
   call mulmul(ia, iu, ie, isga, isgu, isge, ika, iku,
*       ike,nkmax,mhalf)
   if(iwi2.eq.mfull) iwi2 = 0
   call decomp(io, isgo, iwi2, mhalf)
   isgo = -isgo
   iko = 2
   call muldif(ie, io, ia, isge, isgo, isga, ike, iko, ika,
*       nkmax,mhalf)
  endif
  return
  end


## 4. Multiplying Symbolic Polynomial Representations

Given the polynomial decompositions *isga*, *ika*, *ia*(*k*), *k* = 1, …, *ika*, and *isgb*, *ikb*, *ib*(*k*), *k* = 1, …, *ikb*, of two integers *K*_1_ and *K*_2_, respectively, the following is a Fortran subroutine called mulmul that produces the polynomial decomposition *isgo*, *iko*, *io*(*k*), *k* = 1, …, *iko*, of the integer *K*_1_ · *K*_2_ by multiplying the symbolic polynomial representation of *K*_1_ by that of *K*_2_ (as polynomials) to produce a symbolic polynomial representation of *K*_1_ · *K*_2_ from which the polynomial decomposition of *K*_1_ · *K*_2_ can be obtained. Here *nkmax* is the dimension of the arrays *ia*, *ib*, *io* in the calling routine and *mhalf* equals 2^15^. It is noted that the value of *mhalf* is of importance here since given integers *i*, *j*, 1 ≤ *i* ≤ *ika*, 1 ≤ *j* ≤ *ikb*, then 0 ≤ *ia*(*i*) < 2^15^, 0 ≤ *ib*(*j*) < 2^15^, so that the product *ia*(*i*) · *ib*(*j*) is less than 2^30^ and therefore can be stored in a 32 bit integer word.


  subroutine mulmul(ia, ib, io, isga, isgb, isgo, ika, ikb, iko,
*         nkmax,mhalf)
  integer ia(*), ib(*), io(*)
  integer isga, isgb, isgo, ika, ikb, iko, nkmax, mhalf
  integer i, ipt, ipr, iko1, k, j
  if(isga.eq.0.or.isgb.eq.0)then
   isgo=0
   iko = 2
   io(1) = 0
   io(2) = 0
   return
  endif
  iko = ika + ikb
  if(iko.gt.nkmax) stop 110
  if(isga.gt.0)then
   if(isgb.gt.0)then
     isgo = 1
   else
     isgo =-1
   endif
  else
   if(isgb.gt.0)then
    isgo =-1
   else
    isgo = 1
   endif
  endif
   iko1 = iko - 1
   ipr = 0
   do 200 i = 1, iko1
   ipt = ipr
   k = i
   do 180 j = 1, ikb
   if(k .lt. 1) go to 190
   if(k .gt. ika) go to 150
   ipt = ipt + ia(k)*ib(j)
150   continue
   k = k - 1
180   continue
190   continue
   ipr = ipt/mhalf
   io(i) = ipt - ipr*mhalf
200   continue
   io(iko) = ipr
   if(ipr.ge.mhalf) stop 120
   iko1 = iko
   do 300 i = iko1, ika+1, -1
   if(io(i) .ne. 0) go to 400
   iko = iko - 1
300   continue
400   continue
   return
   end


## 5. Subtracting Symbolic Polynomial Representations

Given the polynomial decompositions *isga*, *ika*, *ia*(*k*), *k* = 1, …, *ika*, and *isgb*, *ikb*, *ib*(*k*), *k* = 1, …, *ikb*, of two integers *K*_1_ and *K*_2_, respectively, the following is a Fortran subroutine called muldif that produces the polynomial decomposition *isgo*, *iko*, *io*(*k*), *k* = 1, …, *iko*, of the integer *K*_1_ − *K*_2_ by subtracting the symbolic polynomial representation of *K*_2_ from that of *K*_1_ (as polynomials) to produce a symbolic polynomial representation of *K*_1_ − *K*_2_ from which the polynomial decomposition of *K*_1_ − *K*_2_ can be obtained. Here *nkmax* is the dimension of the arrays *ia*, *ib*, *io* in the calling routine and *mhalf* equals 2^15^. It is noted that by setting *isgb* equal to −*isgb* the polynomial decomposition of *K*_1_ + *K*_2_ can also be obtained with this subroutine.


  subroutine muldif(ia, ib, io, isga, isgb, isgo, ika, ikb, iko,
*          nkmax,mhalf)
  integer ia(*), ib(*), io(*)
  integer isga, isgb, isgo, ika, ikb, iko, nkmax, mhalf
  integer i, iko1, irel
  if(isgb.eq.0)then
   if(isga.eq.0)then
    isgo=0
    iko = 2
    io(1) = 0
    io(2) = 0
    return
   endif
   isgo = isga
   iko = ika
   do 100 i=1,iko
    io(i) = ia(i)
100   continue
  elseif(isga.eq.0)then
   isgo =-isgb
   iko = ikb
   do 200 i=1,iko
    io(i) = ib(i)
200   continue
  else
   iko = ika
   if(ikb.lt.ika) then
    do 300 i=ikb+1,ika
     ib(i) = 0
300  continue
   elseif(ika.lt.ikb) then
    iko = ikb
    do 400 i=ika+1,ikb
     ia(i) = 0
400  continue
   endif
   if(isga*isgb.gt.0)then
    irel = 0
    do 500 i = iko, 1, -1
     if(ia(i).gt.ib(i))then
      irel = 1
      go to 600
    elseif(ia(i).lt.ib(i))then
      irel = -1
      go to 600
    endif
500  continue
600  continue
    if(irel.eq.0)then
     isgo = 0
     do 700 i=1,iko
      io(i) = 0
700     continue
    else
     isgo=isga*irel
     io(1) = (ia(1)-ib(1))*irel
     do 800 i=2,iko
      if(io(i-1).lt.0) then
       io(i) =-1
       io(i-1) = io(i-1) + mhalf
      else
       io(i) = 0
      endif
      io(i) = io(i) + (ia(i)-ib(i))*irel
800     continue
     if(io(iko).lt.0) stop 210
    endif
  else
    isgo=isga
    io(1) = ia(1)+ib(1)
    do 900 i=2,iko
     if(io(i-1).ge.mhalf) then
      io(i) = 1
      io(i-1) = io(i-1) - mhalf
     else
     io(i) = 0
     endif
     io(i) = io(i) + ia(i)+ib(i)
900  continue
     if(io(iko).ge.mhalf) then
     iko = iko+1
     if(iko.gt.nkmax) stop 220
     io(iko) = 1
     io(iko-1) = io(iko-1) - mhalf
   endif
  endif
 endif
 if(iko .eq. 2) go to 990
 iko1 = iko
 do 950 i = iko1, 3, -1
  if(io(i) .ne. 0) go to 990
  iko = iko - 1
950continue
990continue
   return
   end


## 6. Application: Locating a Point Relative to a Plane

Given an integer *n*, *n* ≥ 4, let *S* be a set of *n* points in 3-dimensional space. Given an integer *l*, 0 ≤ *l* ≤ 9, let *x_i_*, *y_i_*, *z_i_*, *i* = 1, …, *n*, be the input decimal coordinates of the points in *S*, and assume that their double precision floating point representations have been rounded off at the *l*th digit of their decimal fractions through applications, one per coordinate, of the two-integer decomposition process. Accordingly, let *x*(*i*), *ix*(*i*), *ix*2(*i*), *y*(*i*), *iy*(*i*), *iy*2(*i*), *z*(*i*), *iz*(*i*), *iz*2(*i*), *i* = 1, …, *n*, be the arrays produced by the three applications of the two-integer decomposition process that contain the rounded off *x*-, *y*-, *z*-coordinates and their two-integer decompositions.

Given points *p*_1_, *p*_2_, *p*_3_ in *S* that are vertices of a non-degenerate triangle, a fundamental problem in computational geometry is that of finding the location of a point *p*_4_ in *S* relative to the plane *H* that contains the triangle. Let *H*^+^ be the open half-space defined by *H* for which *p*_1_, *p*_2_, *p*_3_ appear in a counterclockwise direction around the boundary of the triangle when looking at the triangle from *H*^+^. Let *H*^−^ be the other half-space defined by *H*. Determining in which of *H*, *H*^+^, *H*^−^, the point *p*_4_ is located may not on occasion be satisfactorily done using floating point arithmetic. Accordingly, the following is a Fortran subroutine called crsinn for doing this using polynomial decompositions. On output the sign *isgo* (−1, 0, 1) of some polynomial decomposition determines the location of *p*_4_ (*H^−^*, *H*, *H*^+^).

This routine actually does more. It produces polynomial decompositions *isgox*, *ikox*, *iox*(*k*), *k* = 1, …, *ikox*, *isgoy*, *ikoy*, *ioy*(*k*), *k* = 1, …, *ikoy*, *isgoz*, *ikoz*, *ioz*(*k*), *k* = 1, …, *ikoz*, of integers that are the coordinates of a vector *v* pointing into *H*^+^ and perpendicular to *H*. It also produces the polynomial decomposition *isgo*, *iko*, *io*(*k*), *k* = 1, …, *iko*, of an integer whose sign *isgo* determines the location of *p*_4_ and whose value when divided by both 10*^l^* and the length of *v* is the perpendicular distance from *p*_4_ to *H*. Here *mhalf* equals 2^15^, *mfull* equals 2^30^, and *if ir*, *isec*, *ithi*, *ifou* are locations in the arrays *ix*, *ix*2, etc. corresponding to the points *p*_1_, *p*_2_, *p*_3_, *p*_4_, respectively. In addition, *isclp*(*k*), *k* = 1, 2, is an array such that the polynomial decomposition of 10*^l^* is *isclp*(1), *isclp*(2) (the sign of 10*^l^* and the dimension of array isclp are already known to be 1 and 2, respectively).


  subroutine crsinn(ix, iy, iz, ix2, iy2, iz2, ifir, isec, ithi,
*        ifou, mhalf, mfull, isclp, io, isgo, iko, iox,
*        isgox, ikox, ioy, isgoy, ikoy, ioz, isgoz, ikoz)
  integer ix(*), iy(*), iz(*), ix2(*), iy2(*), iz2(*)
  integer io(*), iox(*),ioy(*), ioz(*)
  integer ifir, isec, ithi, ifou
  integer isclp(*), mhalf, mfull, nkmax
  parameter (nkmax = 30)
  integer iu(nkmax), iv(nkmax), iw(nkmax)
  integer ixt(nkmax), iyt(nkmax), izt(nkmax)
  integer ix3(nkmax), iy3(nkmax), iz3(nkmax)
  integer ix4(nkmax), iy4(nkmax), iz4(nkmax)
  integer ixf(nkmax), iyf(nkmax), izf(nkmax)
  integer ixfiw, iyfiw, izfiw, ixsew, iysew, izsew
  integer ixthw, iythw, izthw, ixfow, iyfow, izfow
  integer ixfi2, iyfi2, izfi2, ixse2, iyse2, izse2
  integer ixth2, iyth2, izth2, ixfo2, iyfo2, izfo2
  integer isgxf, isgyf, isgzf, ikxf, ikyf, ikzf
  integer isgx2, isgy2, isgz2, ikx2, iky2, ikz2
  integer isgx3, isgy3, isgz3, ikx3, iky3, ikz3
  integer isgx4, isgy4, isgz4, ikx4, iky4, ikz4
  integer isgo, iko, isgox, ikox, isgoy, ikoy, isgoz, ikoz
  integer isgu, isgv, isgw, iku, ikv, ikw
  ixfiw = ix(ifir)
  iyfiw = iy(ifir)
  izfiw = iz(ifir)
  ixsew = ix(isec)
  iysew = iy(isec)
  izsew = iz(isec)
  ixthw = ix(ithi)
  iythw = iy(ithi)
  izthw = iz(ithi)
  ixfow = ix(ifou)
  iyfow = iy(ifou)
  izfow = iz(ifou)
  ixfi2 = ix2(ifir)
  iyfi2 = iy2(ifir)
  izfi2 = iz2(ifir)
  ixse2 = ix2(isec)
  iyse2 = iy2(isec)
  izse2 = iz2(isec)
  ixth2 = ix2(ithi)
  iyth2 = iy2(ithi)
  izth2 = iz2(ithi)
  ixfo2 = ix2(ifou)
  iyfo2 = iy2(ifou)
  izfo2 = iz2(ifou)
  call decmp2(ixf, isgxf, ikxf, ixfiw, ixfi2, mhalf, mfull, isclp)
  call decmp2(iyf, isgyf, ikyf, iyfiw, iyfi2, mhalf, mfull, isclp)
  call decmp2(izf, isgzf, ikzf, izfiw, izfi2, mhalf, mfull, isclp)
  call decmp2(io, isgo, iko, ixsew, ixse2, mhalf, mfull, isclp)
  call muldif(io, ixf, ixt, isgo, isgxf, isgx2, iko, ikxf, ikx2,
*      nkmax,mhalf)
  call decmp2(io, isgo, iko, iysew, iyse2, mhalf, mfull, isclp)
  call muldif(io, iyf, iyt, isgo, isgyf, isgy2, iko, ikyf, iky2,
*      nkmax,mhalf)
  call decmp2(io, isgo, iko, izsew, izse2, mhalf, mfull, isclp)
  call muldif(io, izf, izt, isgo, isgzf, isgz2, iko, ikzf, ikz2,
*      nkmax,mhalf)
  call decmp2(io, isgo, iko, ixthw, ixth2, mhalf, mfull, isclp)
  call muldif(io, ixf, ix3, isgo, isgxf, isgx3, iko, ikxf, ikx3,
*      nkmax,mhalf)
  call decmp2(io, isgo, iko, iythw, iyth2, mhalf, mfull, isclp)
  call muldif(io, iyf, iy3, isgo, isgyf, isgy3, iko, ikyf, iky3,
*      nkmax,mhalf)
  call decmp2(io, isgo, iko, izthw, izth2, mhalf, mfull, isclp)
  call muldif(io, izf, iz3, isgo, isgzf, isgz3, iko, ikzf, ikz3,
*      nkmax,mhalf)
  call decmp2(io, isgo, iko, ixfow, ixfo2, mhalf, mfull, isclp)
  call muldif(io, ixf, ix4, isgo, isgxf, isgx4, iko, ikxf, ikx4,
*      nkmax,mhalf)
  call decmp2(io, isgo, iko, iyfow, iyfo2, mhalf, mfull, isclp)
  call muldif(io, iyf, iy4, isgo, isgyf, isgy4, iko, ikyf, iky4,
*      nkmax,mhalf)
  call decmp2(io, isgo, iko, izfow, izfo2, mhalf, mfull, isclp)
  call muldif(io, izf, iz4, isgo, isgzf, isgz4, iko, ikzf, ikz4,
*      nkmax,mhalf)
  call mulmul(iyt, iz3, iv, isgy2, isgz3, isgv, iky2, ikz3, ikv,
*      nkmax,mhalf)
  call mulmul(izt, iy3, iu, isgz2, isgy3, isgu, ikz2, iky3, iku,
*      nkmax,mhalf)
  call muldif(iv, iu, iox, isgv, isgu, isgox, ikv, iku, ikox,
*      nkmax,mhalf)
  call mulmul(iox, ix4, io, isgox, isgx4, isgo, ikox, ikx4, iko,
*      nkmax,mhalf)
  call mulmul(izt, ix3, iv, isgz2, isgx3, isgv, ikz2, ikx3, ikv,
*      nkmax,mhalf)
  call mulmul(ixt, iz3, iu, isgx2, isgz3, isgu, ikx2, ikz3, iku,
*      nkmax,mhalf)
  call muldif(iv, iu, ioy, isgv, isgu, isgoy, ikv, iku, ikoy,
*      nkmax,mhalf)
  call mulmul(ioy, iy4, iu, isgoy, isgy4, isgu, ikoy, iky4, iku,
*      nkmax,mhalf)
  isgu =-isgu
  call muldif(io, iu, iw, isgo, isgu, isgw, iko, iku, ikw,
*      nkmax,mhalf)
  call mulmul(ixt, iy3, iv, isgx2, isgy3, isgv, ikx2, iky3, ikv,
*      nkmax,mhalf)
  call mulmul(iyt, ix3, iu, isgy2, isgx3, isgu, iky2, ikx3, iku,
*      nkmax,mhalf)
  call muldif(iv, iu, ioz, isgv, isgu, isgoz, ikv, iku, ikoz,
*      nkmax,mhalf)
  call mulmul(ioz, iz4, iu, isgoz, isgz4, isgu, ikoz, ikz4, iku,
*      nkmax,mhalf)
  isgu =-isgu
  call muldif(iw, iu, io, isgw, isgu, isgo, ikw, iku, iko,
*      nkmax,mhalf)
  return
  end


Sometimes besides knowing the location of the point *p*_4_ relative to the plane *H* it may be desirable to know the perpendicular distance from *p*_4_ to *H*. The following is Fortran code for this purpose. It uses the polynomial decompositions that are part of the output of subroutine crsinn. Variables here are either integer or double precision following convention. Here *r*215 equals (2.0*d*0)^15^, *dscle* equals (10.0*d*0)*^l^*, and *dist* is the resulting signed perpendicular distance. In addition, it is assumed that subroutine doubnm (presented below) exists for transforming the polynomial decomposition of an integer into the double precision floating point value of the integer.


  call crsinn(ix, iy, iz, ix2, iy2, iz2, ifir, isec, ithi, ifou,
*       mhalf, mfull, isclp, io, isgo, iko, iox, isgox,
*       ikox, ioy, isgoy, ikoy, ioz, isgoz, ikoz)
  call doubnm(io, isgo, iko, r215, dnum)
  call doubnm(iox, isgox, ikox, r215, xnum)
  call doubnm(ioy, isgoy, ikoy, r215, ynum)
  call doubnm(ioz, isgoz, ikoz, r215, znum)
  dnux = dmax1(dabs(xnum),dabs(ynum),dabs(znum))
  xnum = xnum/dnux
  ynum = ynum/dnux
  znum = znum/dnux
  dnom = dsqrt(xnum**2+ynum**2+znum**2)
  dist = ((dnum/dnux)/dnom)/dscle


The following is subroutine doubnm that was called above.


  subroutine doubnm(io, isgo, iko, r215, dnum)
  integer io(*)
  double precision dnum, r215, rpwr
  integer isgo, iko, i
  if(isgo.eq.0) then
    dnum = 0.0d0
    go to 900
  else
    if(iko .lt. 2) stop 310
    if(iko .gt. 68) stop 320
    rpwr = 1.0d0
    dnum = dble(io(1))
    do 100 i = 2, iko
       rpwr = rpwr*r215
       dnum = dnum + dble(io(i))*rpwr
100    continue
  endif
  if(isgo.lt.0) dnum = -dnum
900 continue
  return
  end


## 7. Numerical Examples

Twelve lines follow, each line containing three numbers. Each line corresponds to a point in 3-dimensional space, and the three numbers in the line correspond to the *x*-, *y*-, *z*-coordinates of the point, in that order. Given *i*, 1 ≤ *i* ≤ 12, point *i* is the point corresponding to the *i*th line. It is assumed that the coordinates of the twelve points are read into double precision arrays *x*(*i*), *y*(*i*), *z*(*i*), *i* = 1, …, 12, so that *x*(*i*), *y*(*i*), *z*(*i*) contain the *x*-, *y*-, *z*-coordinates, respectively, of point *i*.

**Table:** 

−13.729277089	14.530621914	97.981467003
38.000000000	7.049967880	−92.123710427
41.736468803	68.831641719	−59.331882431
85.557213025	−49.840807038	−13.994897166
33.675274550	−77.937397763	52.741164465
1.724283838	−53.594476834	−84.424190762
15.161728368	3.186043237	98.792566086
0.082570927	−30.956721161	−95.085758310
47.541325082	−77.446759923	−41.735139045
−33.285508962	−14.545102894	93.175307798
−2.277195916	−58.886394970	80.791131020
70.061142979	9.068097315	−70.800333278

Given *l* equal to 8, through the two-integer decomposition process, the numbers above are rounded off at the *l*th = 8th digit of their decimal fractions and saved in *x*(*i*), *y*(*i*), *z*(*i*), *i* = 1, …, 12, so that then they appear as follows.

**Table:** 

−13.729277090	14.530621910	97.981467000
38.000000000	7.049967880	−92.123710430
41.736468800	68.831641720	−59.331882430
85.557213020	−49.840807040	−13.994897170
33.675274550	−77.937397760	52.741164460
1.724283840	−53.594476830	−84.424190760
15.161728370	3.186043240	98.792566090
0.082570930	−30.956721160	−95.085758310
47.541325080	−77.446759920	−41.735139040
−33.285508960	−14.545102890	93.175307800
−2.277195920	−58.886394970	80.791131020
70.061142980	9.068097310	−70.800333280

Each rounded off coordinate is also decomposed into two integers. Twelve lines follow, each line containing six integers. For each *i*, *i* = 1, …, 12, the first two integers in the *i*th line are the two integers into which the *x*-coordinate of point *i* is decomposed. Similarly the next two integers correspond to the *y*-coordinate, and the last two to the *z*-coordinate. The two-integer decompositions of the twelve points are then saved into *ix*(*i*), *ix*2(*i*), *iy*(*i*), *iy*2(*i*), *iz*(*i*), *iz*2(*i*), *i* = 1, …, 12, in the obvious manner. It is noted that when *mfull* = 2^30^ = 1073741824 appears as the second integer corresponding to a coordinate it is to be interpreted as a zero.

**Table:** 

−13	−72927709	14	53062191	97	98146700
38	1073741824	704996788	0	−92	−12371043
41	73646880	68	83164172	−59	−33188243
85	55721302	−49	−84080704	−13	−99489717
33	67527455	−77	−93739776	52	74116446
172428384	0	−53	−59447683	−84	−42419076
15	16172837	318604324	0	98	79256609
8257093	0	−30	−95672116	−95	−8575831
47	54132508	−77	−44675992	−41	−73513904
−33	−28550896	−14	−54510289	93	17530780
−227719592	0	−58	−88639497	80	79113102
70	6114298	906809731	0	−70	−80033328

Given *l* as above equal to 8 and setting *isclu* to 10*^l^* = 10^8^, by calling subroutine decomp the polynomial decomposition of *isclu* is found to be *isgcl*, *isclp*(1), *isclp*(2), with *isgcl* equal to 1, *isclp*(1) equal to 24832 and *isclp*(2) equal to 3051 (10^8^ = 24832 + 3051 · *mhalf* = 24832 + 3051 · 2^15^).

As an example of how exact computations are carried out that reference coordinates of the input points given above, the computation that is the product of the *x*-coordinate of point 2 and the *y*-coordinate of point 4 minus the product of the *y*-coordinate of point 2 and the *x*-coordinate of point 4 is described. Using rounded-off numbers the result of the computation should equal *x*(2) · *y*(4) − *y*(2) · *x*(4), i. e.,


(38.000000000) · (−49.840807040) − (7.049967880) · (85.557213020).


On the other hand, if exact computations are required then each of the four numbers involved must first be converted into an integer which is the number times 10^8^ rounded off at the decimal point. Since the resulting integer may be too big to be saved into a 32 bit integer word, its polynomial decomposition in the form of two or more 32 bit integer words is obtained instead. Thus, for example, the polynomial decomposition of the integer which is the *x*-coordinate of point 4 times 10^8^ rounded off at the decimal point can be obtained by calling subroutine decmp2 using the two-integer decomposition of the coordinate, i.e. the integers 85 and 55721302, and the polynomial decomposition of 10^8^ as obtained above. The resulting polynomial decomposition is then found to be *isgox*4, *ikox*4, *iox*4(*k*), *k* = 1, …, *ikox*4, with *isgox*4 equal to 1, *ikox*4 equal to 3, *iox*4(k), *k* = 1, 2, 3, equal to 29270, 31723, and 7 (the integer which is the *x*-coordinate of point 4 times 10^8^ rounded off at the decimal point equals 29270 + 31723 · (2^15^) + 7 · (2^15^)^2^). In the same manner the polynomial decompositions associated with the *x*-, *y*-coordinates of point 2, and the *y*-coordinate of point 4 are found to be, respectively, *isgox*2, *ikox*2, *iox*2(*k*), *k* = 1, …, *ikox*2, *isgoy*2, *ikoy*2, *ioy*2(k), *k* = 1, …, *ikoy*2, *isgoy*4, *ikoy*4, *ioy*4(*k*), *k* = 1, …, *ikoy*4, with *isgox*2 equal to 1, *ikox*2 equal to 3, *iox*2(*k*), *k* = 1, 2, 3, equal to 26112, 17662, 3, *isgoy*2 equal to 1, *ikoy*2 equal to 2, *ioy*2(*k*), *k* = 1, 2, equal to 26036, 21514, *isgoy*4 equal to −1, *ikoy*4 equal to 3, *ioy*4(*k*), *k* = 1, 2, 3, equal to 2368, 21030, 4. Finally, using these polynomial decompositions as input the desired result is obtained by calling subroutines mulmul and muldif as follows. Here *nkmax* is the dimension of all of the arrays (input and output).


call mulmul(iox2,ioy4,iu,isgox2,isgoy4,isgu,ikox2,ikoy4,iku,nkmax,mhalf)
call mulmul(ioy2,iox4,iv,isgoy2,isgox4,isgv,ikoy2,ikox4,ikv,nkmax,mhalf)
call muldif(iu,iv,io,isgu,isgv,isgo,iku,ikv,iko,nkmax,mhalf)


The polynomial decomposition of an integer that approximates the desired result times (10^8^)^2^ rounded off at the decimal point is then found to be *isgo*, *iko*, *io*(*k*), *k* = 1, …, *iko*, with *isgo* equal to −1, *iko* equal to 5, *io*(*k*), *k* = 1, …, 5, equal to 21112, 15183, 31880, 21597, 21. The desired result is then approximately equal to this integer, i.e. 21112 + 15183 · (2^15^) + 31880 · (2^15^)^2^ + 21597 · (2^15^)^3^ + 21 · (2^15^)^4^, divided by (10^8^)^2^. By calling subroutine doubnm the integer can be approximated by a double precision number which when divided by (10^8^)^2^ is approximately −2497.1262712133, an approximation of the desired result.

Finally, an example is given for the purpose of describing the process of locating a point relative to a plane that contains three other points. Here the point whose location is desired is point 12 as given above, and the three other points are point 1, point 2, point 8 also as given above. With *t* as the triangle with vertices point 1, point 2, point 8, and *H* as the plane that contains *t*, *H*^+^ is taken to be the open half-space defined by *H* for which point 1, point 2, point 8 appear in a counterclockwise direction around the boundary of *t* when looking at *t* from *H*^+^. *H*^−^ is taken to be the other half-space defined by *H*. With ifir set to 1, isec set to 2, ithi set to 8, ifou set to 12, which of *H*, *H*^+^, *H*^−^ contains point 12 can be determined by calling subroutine crsinn as follows.


  call crsinn(ix, iy, iz, ix2, iy2, iz2, ifir, isec, ithi, ifou,
*       mhalf, mfull, isclp, io, isgo, iko, iox, isgox,
*       ikox, ioy, isgoy, ikoy, ioz, isgoz, ikoz)


On output *isgo* equals −1 so that point 12 must be in *H*^−^. In addition *iko* equals 7, *io*(*k*), *k* = 1, …, 7, equals 21844, 27853, 3870, 5372, 13630, 9887, 213, *isgox* equals −1, *ikox* equals 5, *iox*(*k*), *k* = 1, …, 5, equals 11868, 15341, 2677, 15631, 62, *isgoy* equals 1, *ikoy* equals 5, *ioy*(*k*), *k* = 1, …, 5, equals 11577, 8756, 364, 27887, 63, *isgoz* equals −1, *ikoz* equals 5, *ioz*(*k*), *k* = 1, …, 5, equals 5921, 23919, 26934, 16812, 19. By calling subroutine doubnm the integer whose polynomial decomposition is *isgo*, *iko*, *io*(*k*), *k* = 1, …, *iko*, can be approximated by a double precision number *dnum*. Similarly, the three integers, say *ix*, *iy*, *iz*, whose polynomial decompositions are *isgox*, *ikox*, *iox*(*k*), *k* = 1, …, *ikox*, *isgoy*, *ikoy*, *ioy*(*k*), *k* = 1, …, *ikoy*, *isgoz*, *ikoz*, *ioz*(*k*), *k* = 1, …, *ikoz*, can be approximated, respectively, by double precision numbers *xnum*, *ynum*, *znum*. The vector (*ix*, *iy*, *iz*), which is perpendicular to the plane *H* and points into *H*^+^, can then be approximated by the vector (*xnum*, *ynum*, *znum*). Finally, by dividing *dnum* by both the length of the vector (*xnum*, *ynum*, *znum*) and 10^8^ the number −25.047402554921 is approximately obtained, an approximation of the signed perpendicular distance from point 12 to plane *H*, the negative sign indicating that point 12 is in *H*^−^.

## 8. Summary

A scheme has been presented and software has been documented for transforming into a series of integers input decimal numbers that have been read into a computer as double precision floating point numbers and for carrying out multiplication, addition and subtraction operations based on these numbers using their integer representations. The total number of significant digits of each input number must not be greater than 14, and the number of digits to the left of the decimal point must not exceed 9. Through a preprocessing step the double precision floating point representation of each input decimal number is rounded off at a prespecified location of its decimal fraction, a location no more than 9 digits to the right of the decimal point, and the rounded off number is decomposed into two integers. All operations that follow involving the input number are then carried out in terms of the rounded off double precision floating point number and when this is not satisfactory in terms of the two integers. This scheme has been successfully incorporated into several computational geometry related programs such as REGTET [1]. Other programs that incorporate this scheme can be found at http://math.nist.gov/~JBernal/JBernal_Sft.html. Besides being used for locating a point relative to a plane, in these programs the scheme has also been used for locating a point relative to a sphere, for computing the intersection of a line and a plane, for computing the center of a sphere, etc.
